# GLUT1, GLUT3 Expression and 18FDG-PET/CT in Human Malignant Melanoma: What Relationship Exists? New Insights and Perspectives

**DOI:** 10.3390/cells10113090

**Published:** 2021-11-09

**Authors:** Gerardo Cazzato, Anna Colagrande, Antonietta Cimmino, Caterina Abbatepaolo, Emilio Bellitti, Paolo Romita, Lucia Lospalluti, Caterina Foti, Francesca Arezzo, Vera Loizzi, Teresa Lettini, Sara Sablone, Leonardo Resta, Gennaro Cormio, Giuseppe Ingravallo, Roberta Rossi

**Affiliations:** 1 Section of Pathology, Department of Emergency and Organ Transplantation (DETO), University of Bari, “Aldo Moro”, 70124 Bari, Italy; anna.colagrande@gmail.com (A.C.); micasucci@inwind.it (A.C.); abbatepaolocaterina@libero.it (C.A.); emilio.bellitti@gmail.com (E.B.); teresa.lettini@uniba.it (T.L.); leonardo.resta@uniba.it (L.R.); giuseppe.ingravallo@uniba.it (G.I.); roberta.rossi@policlinico.ba.it (R.R.); 2 Section of Dermatology, Department of Biomedical Sciences and Human Oncology, University of Bari, “Aldo Moro”, 70124 Bari, Italy; paolo.romita@uniba.it (P.R.); l.lospalluti@gmail.com (L.L.); caterina.foti@uniba.it (C.F.); 3 Section of Ginecology and Obstetrics, Department of Biomedical Sciences and Human Oncology, University of Bari, “Aldo Moro”, 70124 Bari, Italy; francesca.arezzo@uniba.it (F.A.); vera.loizzi@uniba.it (V.L.); gennaro.cormio@uniba.it (G.C.); 4 Section of Legal Medicine, Department of Interdisciplinary Medicine, Bari Policlinico Hospital, University of Bari, “Aldo Moro”, 70124 Bari, Italy; sara.sablone@policlinico.ba.it

**Keywords:** GLUT1, GLUT3, malignant melanoma, immunohistochemistry, perspectives

## Abstract

Background: Malignant melanoma is the most aggressive of skin cancers and the 19th most common cancer worldwide, with an estimated age-standardized incidence rate of 2.8–3.1 per 100,000; although there have been clear advances in therapeutic treatment, the prognosis of MM patients with Breslow thickness greater than 1 mm is still quite poor today. The study of how melanoma cells manage to survive and proliferate by consuming glucose has been partially addressed in the literature, but some rather interesting results are starting to be present. Methods: A systematic review was conducted following the Preferred Reporting Items for Systematic Reviews and Meta-Analyses (PRISMA) guidelines and a search of PubMed and Web of Sciences (WoS) databases was performed until 27 September 2021 using the terms: glucose transporter 1 and 3 and GLUT1/3 in combination with each of the following: melanoma, neoplasm and immunohistochemistry. Results: In total, 46 records were initially identified in the literature search, of which six were duplicates. After screening for eligibility and inclusion criteria, 16 publications were ultimately included. Conclusions: the results discussed regarding the role and expression of GLUT are still far from definitive, but further steps toward understanding and stopping this mechanism have, at least in part, been taken. New studies and new discoveries should lead to further clarification of some aspects since the various mechanisms of glucose uptake by neoplastic cells are not limited to the transporters of the GLUT family alone.

## 1. Introduction

Malignant melanoma is the most aggressive of skin cancers and the 19th most common cancer worldwide, with an estimated age-standardized incidence rate of 2.8–3.1 per 100,000 [1]. Although the advent of new targeted molecular therapies (directed against BRAF mutations) and immunotherapy have made it possible to improve the survival and clinical outcome of affected patients, even today the diagnosis of melanoma still has a strong impact on the patient’s life, and survival rates are strictly dependent on independent prognostic markers, above all, Breslow thickness [2,3]. In the diagnostics, staging, restaging and follow-up of patients suffering from various neoplasms, including melanoma, positron emission tomography/CT with the use of 18 fluoro-deoxy-glucose is considered the “gold standard” investigation [4]. The underlying physical principle relates to the notion that proliferating cells modify their glucose metabolism so as to allow them to “feed” through the process of aerobic glycolysis (Warburg effect), surviving and maintaining the neoplastic clone in expansion [5]. Against this background, some works in the literature have shed some light on the potential relationships between glucose uptake, visualization in FDG-PET/CT and predominantly immunohistochemical expression of glucose transporters 1 and 3: that they have a high affinity for glucose and can operate in an insulin-independent manner. In this paper, we conduct a detailed review of the existing works in the literature on this topic, outlining the state of the art in this field and, finally, consider future viable paths.

## 2. Materials and Methods

A systematic review was conducted following the Preferred Reporting Items for Systematic Reviews and Meta-Analyses (PRISMA) guidelines. A search of PubMed and Web of Sciences (WoS) databases was performed until 27 September 2021 using the terms: glucose transporter 1 and 3 and GLUT1/3 in combination with each of the following: melanoma, neoplasm and immunohistochemistry. Only articles in English were selected. Eligible articles were assessed according to the Oxford Centre for Evidence-Based Medicine 2011 guidelines [6]. Review articles, meta-analyses, observational studies and letters to the editor were included. Other potentially relevant articles were identified by manually checking the references of the included literature.

An independent extraction of articles was performed by two investigators according to the inclusion criteria. Disagreement was resolved by discussion between the two review authors. Since the study designs, participants, treatment measures and reported outcomes varied markedly, we focused on describing the different approaches of the authors regarding the expression of GLUT1 and GLUT3 in malignant melanoma, analyzed the techniques (mainly immunohistochemistry) used in the works examined and, finally, analyzed the state of the art, imagining what the future perspectives may be.

## 3. Results

**Figure 1:**
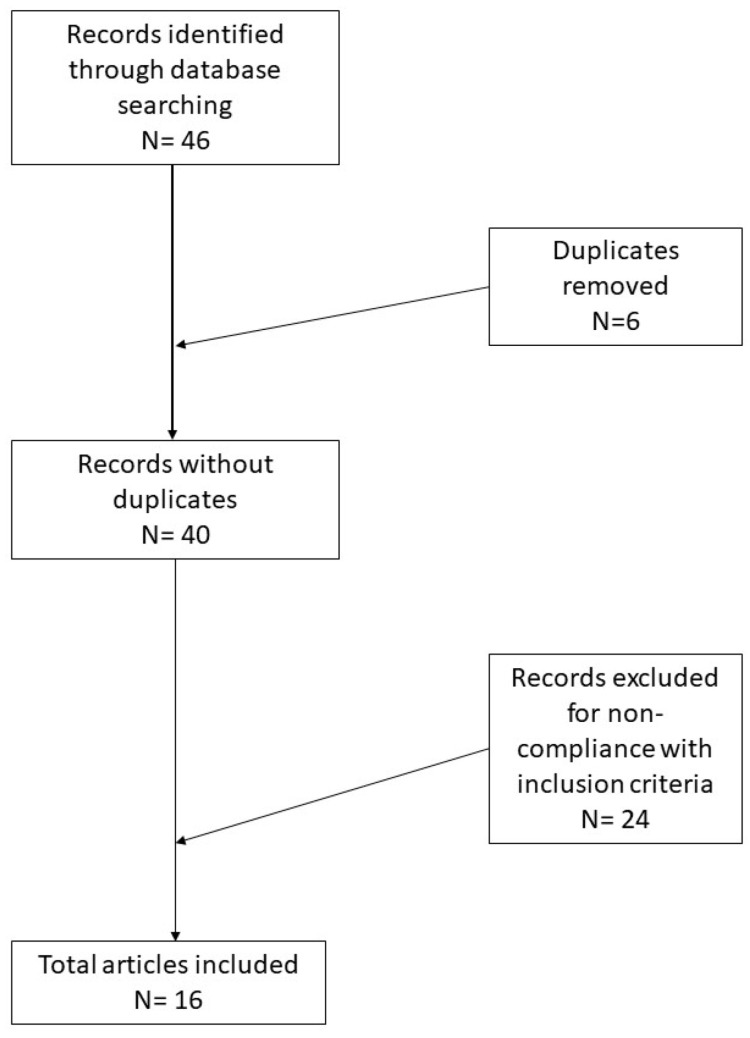
Research of the literature and selection of articles according to PRISMA guidelines.

In total, 46 records were initially identified in the literature search, of which six were duplicates. After screening for eligibility and inclusion criteria, 16 publications were ultimately included (Figure 1). The study and features are summarized in Table 1. Most of the publications were original and/or research articles (*n* = 12), followed by reviews with or without metanalysis (*n* = 2), a comparative study (*n* = 1) and a case–control retrospective study (*n* = 1). All studies included were rated as level 4 or 5 evidence for clinical research as detailed in the Oxford Centre for Evidence-Based Medicine 2011 guidelines [6].

## 4. Discussion

Melanoma continues to be the most aggressive cutaneous malignancy of all [1,23], featuring different incidence and prevalence rates on the various continents, but which tend to remain rather stable [23]. Classically, MM has always been distinguished as cutaneous or mucosal according to the site of onset, but, in recent years, research has made it possible to study in greater depth the mechanisms of the genetic–molecular landscape of both types, offering a better understanding of the points of contact and divergence [7,8]. It has long been known that glucose transporters (GLUT1-14) belong to the family of structurally related proteins that mediate the energy-independent transport of glucose across the plasma membrane. Among the various types, the most historically studied have been GLUT1, expressed mostly in erythrocytes, endothelial cells of the blood–brain barrier and placental cells and GLUT3, also present in various tissues [9,10]. Different works have shown that the basic principle on which the use of FDG-PET/CT is based is the increased energy requirement of cancer cells, with a consequent increase in the expression of GLUT1 and the neoplasm capacity to proliferate, survive and, finally, metastasize. This mechanism has been well studied and characterized in neoplasms of the breast, pancreas, cervix, endometrium, lung, mesothelium, colon, bladder, thyroid, bone, soft tissues and oral cavity [10,11,12]. Conversely, there are conflicting results regarding the role played by GLUT3 in various malignancies [13]. In the field of melanocytic pathology, we probed the role of GLUT1/3 in mediating glucose uptake and correlated findings with the clinical outcome.

In 2002, Wachsberger et al. demonstrated, by Western immunoblotting performed on 31 melanoma biopsies, a wide variability in the expression of both GLUT1 and HK and proposed the explanation that a broad spectrum of transporter activities could differently influence the response of patients to the same therapeutic treatment [14]. Three years later, Yamada et al. [15] conducted a study using four human melanoma cell lines (SK-MEL 23, SK-MEL 24 and G361) and one mouse (B16): the authors demonstrated that factors such as cell viability and rate of cell proliferation, as well as HK expression, were directly responsible for an increased glucose uptake at FDG-PET/CT. However, there did not appear to be any correlation between GLUT1 expression and the degree of glucose uptake. In another study, in 2008, Parente et al. reported results obtained from the analysis of 44 skin lesions, consisting of 12 benign nevi, 12 Spitz nevi and 20 primitive melanomas. The authors conducted an immunohistochemical study for markers such as GLUT1 and GLUT3 and reported that, unlike GLUT3 (expressed in all the lesions analyzed), GLUT1 was downregulated in 55% of primary melanomas, suggesting that glucose transport occurred across the plasma membrane by other transporters [16]. In 2009, Strobel et al. reported their experience with liver metastases resection samples from uveal and skin melanoma. Studying the reliability of FDG-PET/CT and the serum S-100B assay, the authors found that, while these determinations were reliable (sensitive) for skin-priming melanoma metastases, they were not as reliable in cases of metastasis from uveal melanoma [17]. In 2012, Park et al. conducted a detailed study to investigate the correlative association between glucose uptake in FDG-PET/CT, GLUT1/3 expression, exokinase-2 (HK-2) expression and Ki-67 expression in malignant melanoma. Enrolling 19 patients with a proven histological diagnosis of MM and pre-subjected to FDG-PET/CT, the authors found that the metabolic uptake increased with increasing GLUT1/3 expression, while HK-2 and Ki67 did not appear to be correlated [18,19,20]. Another important aspect was addressed by two other groups [21,24] regarding the role that hypoxia plays in the translocation and, therefore, expression of GLUT in cancer cells: from these works it emerged that the hypoxic condition is able to activate a pathway signal transduction mediated by HIF-1-alpha. In addition to other actions, this is able to promote the expression of glucose transporters, allowing the neoplastic cell to continue to survive through airborne glycolysis (Warburg effect) from oxidative phosphorylation. This has also been partially demonstrated in non-melanoma skin cancer (NMSC) [24].

In 2019, Dura et al. conducted a study of 400 cases (225 melanomas and 175 benign nevi) to evaluate the expression of GLUT1 in immunohistochemistry. They found that 69/225 melanomas were positive for this marker and, moreover, demonstrated an increasing expression (evaluated according to a semiquantitative score, Immunoscore 15) with increasing Breslow thickness. Intriguingly, the authors demonstrated that GLUT1 expression was correlated with shorter 10-year disease-free survival, relapse-free survival and shorter metastasis-free survival (MFS) [25]. Various other authors [22,26,27,28,29] have examined these aspects, sometimes with confirmatory but sometimes contradictory results. A very interesting recent paper by Reckzeh et al. described the discovery of a powerful inhibitor of glucose transporters 1 and 3, Glutor, that is capable of blocking the uptake of neoplastic cells, blocking the “metabolic plasticity” of cancer cells. Used together with a CB-839 glutaminase inhibitor, the combination can block cell proliferation and survival [30].

Importantly, a certain variability in the results obtained from immunohistochemistry for GLUT1-3 emerges from this revision of the literature. It is necessary to bear in mind that in immunoassays the binding between antibody and protein (GLUT) could be hindered by the phenomenon of the glycosylation of these receptors, following which they become more similar to glucose [31]. Finally, future research directions should take into account the different subcellular localization of GLUTs depending on the various isoforms and the various functional moments [32].

## 5. Conclusions

A better knowledge and closer study of the tumor phenotype have been for many years one of the most important targets in cancer research. The study of how the cancer cell is able to evade control mechanisms and apoptosis and proliferate has aroused great interest and attention. Against this background, various efforts have been made to better understand the metabolism of neoplastic cells, paying particular attention to the concept of “metabolic plasticity” and the Warburg effect. The results discussed regarding the role and expression of GLUT are still far from definitive, but further steps toward understanding and stopping this mechanism have, at least in part, been taken. New studies and new discoveries should lead to the further clarification of some aspects since the various mechanisms of glucose uptake by neoplastic cells are not limited to the transporters of the GLUT family alone.

## Figures and Tables

**Table 1:** Summary of the papers examined in this review.

Author(s)	Type of Paper	Neoplasm	Procedure	Results
Park et al. [7]	Research article	19 cases of malignant melanoma	FDG-PET/CT and immunohistochemistry for GLUT1/3	GLUT1/3 mediate glucose uptake in FDG-PET/CT HK-2 and Ki-67 play no role
Dura et al. [8]	Original article	225 cases of MM/175 benign nevus	Immunohistochemistry for GLUT1	GLUT1 was expressed in 69/225 MM GLUT1 was negative in benign nevus
Strobel et al. [9]	Research article	27 liver metastases of 13 patients with uveal melanoma 43 liver metastases of 14 patients with CMM	FDG-PET/CT S100B serum marker Cytology Histology Immunohistochemistry for GLUT1 and S-100	46% of liver metastases by UM were negative in FDG- PET/CT All liver metastases by CMM were positive S-100B values were significantly higher in the CM patient group compared with the UM patients No obvious difference for GLUT1 and S-100 protein
Ruby et al. [10]	Research article	91 MM 18 metastases of MM 56 benign nevus	Immunohistochemistry for GLUT2/3	GLUT2 was negative in all melanomas and benign nevi 85.3% expressed either GLUT1 or GLUT3 or both, 39.4% of melanoma cases coexpressed GLUT1 and GLUT3, 17.4% of melanoma cases only expressed GLUT1, 28.4% of melanoma cases only expressed GLUT3 and 14.7% of melanoma cases were negative for both markers
Seleit et al. [11]	Case–control and retrospective study	20BCC 20 SCC	Immunohistochemistry for HIF-1-alpha and GLUT1	HIF-1-alpha has a role in NMSC pathogenesis, GLUT1 is downregulated
Wachsberger et al. [12]	Original article	31 MM	Western immunoblot analysis for GLUT1 and HK-2	30 MM exhibited a 22-fold variation in levels of GLUT1 and 29 exhibited a nine-fold variation in total cellular Hexokinase-II activity
Slominski et al. [13]	Original article	2 cellular lines of MM: amelanotic/melanotic	Cultured cells	Melanogenesis upregulates HIF-1-alpha expression in MM
Na et al. [14]	Original article	63 pulmonary squamous cell carcinoma	FDG-PET/CT RNA-seq Immunohistochemistry	GLUT1/3 may be responsible for a different response to immunotherapy
Yamada et al. [15]	Comparative study	4 cellular lines of human MM: SK-MEL 23, SK-MEL 24 and G361 and 1 murine: B16	FDG-PET/CT Immunohistochemistry for HK-2 and GLUT1	Proliferation rate, cell viability and HK expression were important to mediate FDG uptake
Parente et al. [16]	Original article	Malignant melanoma/benign nevus	Immunohistochemistry for GLUT1-3	GLUT1: positive in >90% benign nevus GLUT1: absent in 55% MM GLUT3: present in both (MM and benign nevus)
Singer et al. [17]	Research article	249 RCC	Tissue microarray analysis	In RCC an increased expression of GLUT1 correlates with a decreased presence of CD8 + T lymphocytes
Airley et al. [18]	Research article	Different and various tumors	Tissue microarray analysis and immunohistochemistry for GLUT1	Glut-1 expression in peri- necrotic regions
Meyer et al. [19]	Meta-analysis	Different and various tumors	FDG-PET/TC	Overall, only a moderate association was found between GLUT 1/3 expression and SUV values derived from FDG-PET
Chen et al. [20]	Review	12 studies about GLUT1 2 studies about GLUT3	Review with meta-analysis	A combination of GLUTs 1 and 3 might help predict malignancy of cancers and direct effective cancer therapy
Reckzeh et al. [21]	Original article	Various cellular lines	Various procedures	Glutor, very potent glucose uptake inhibitor
Su et al. [22]	Research article	Cellular line of MM: A375	Immunoprecipitation and siRNA	CD147 could downregulate GLUT1 expression by reducing the proliferative rate of MM cells

## Data Availability

Not applicable.
